# Simultaneous Occurrence of Duane Retraction Syndrome with Marfan Syndrome

**DOI:** 10.1155/2011/784259

**Published:** 2011-12-22

**Authors:** Mihir Kothari, Florence Manurung, Bhavesh Mithiya

**Affiliations:** ^1^Department of Pediatric Ophthalmology and Strabismus, Aditya Jyot Eye Hospital, Mumbai 400 031, India; ^2^Department of Pediatric Ophthalmology, Jyotirmay Eye Clinic, Thane 400 601, India; ^3^Department of Pediatrics and Department of Pediatric Ophthalmology, Jupiter Hospital, Thane 400 601, India

## Abstract

Marfan syndrome (MFS) is an autosomal dominant disorder of connective tissue, while Duane retraction syndrome (DRS) is a congenital cranial dysinnervation disorder (CCDD) which can be transmitted as autosomal dominant disorder in 5–10% of patients. In this paper, we present an 8-year-old girl who presented with left eye DRS and bilateral subluxation of the lens associated with MFS in absence of familial involvement. To our knowledge this is the first case report of DRS with MFS. The occurrence of these syndromes together is very rare and appears to be coincidental.

## 1. Introduction

Marfan syndrome (MFS) is an autosomal dominant disorder of connective tissue due to mutation of the fibrillin gene [1]. Duane retraction syndrome (DRS) is a congenital cranial dysinnervation disorder (CCDD), usually not inherited but can be transmitted as autosomal dominant disorder in 5–10% patients [2].

In this paper, we present an 8-year-old girl who presented with left eye DRS and bilateral subluxation of the lens associated with MFS. To our knowledge this is the first case report of DRS with MFS.

## 2. Case Report

An 8-year-old girl presented with blurred vision in both eyes and an abnormal head posture. Her birth history, systemic history, and family history was nonremarkable. The parents and the elder brother were normal. There was no history of consanguinity. Ophthalmic examination revealed best corrected visual acuity (BCVA) of 20/80 in each eye on Snellen's 6 meter logMAR chart with −7.00–1.00 × 180 in the right eye and −8.00–1.50 × 120 in the left eye. Her near vision with the distance correction was 20/125 on Richmond's near vision chart at 40 cm which improved to 20/30 with +3D addition in each eye.

**Figure 1:**
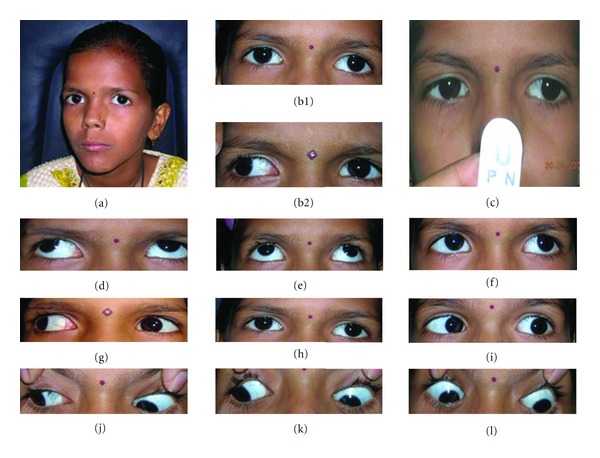
Digital face photographs of the patient demonstrating (a) orthotropia with right face turn, (b1) left eye exotropia in forced primary position when fixing with the right eye, (b2) increase in the deviation while fixing with the left eye, (c) absence of convergence, (d–l) ocular movements in cardinal positions of gaze.

Orthoptic examination (Figure 1) was significant for a 20-degree right side face turn with which she had orthotropia for the near and distance. In forced primary position, she had a 20-degree exotropia in the left eye. The exotropia was 45 degrees while fixing with the left eye. The exotropia increased in upgaze and reduced in downgaze (V pattern). Abduction and adduction in the left eye were limited. The extraocular movements in the right eye were normal. There was no globe retraction, palpebral fissure changes, upshoot or downshoot in the left eye. Convergence was absent.

**Figure 2:**
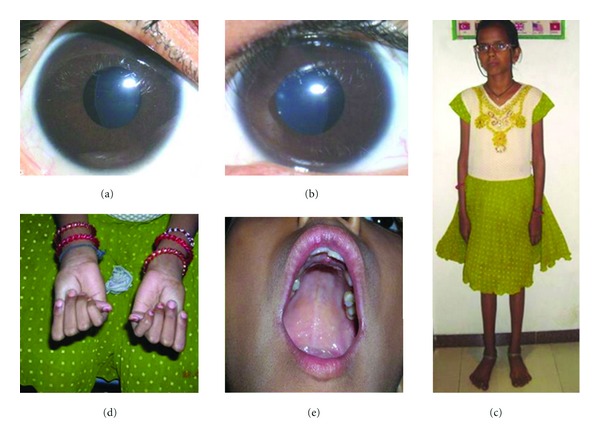
Features suggestive of MFS. (a) Nasal subluxation of crystalline lens in the right eye and (b) in the left eye, (c) tall and thin stature with long arms, (d) positive thumb sign and arachnodactyly, (e) high arched palate with crowding of teeth.

Slit lamp biomicroscopy revealed deep anterior chambers with nasal subluxation of the crystalline lens in both eyes (Figure 2). The equator of the lenses was visible in the pupillary area dividing the pupil in small aphakic (10–20%) area and in large phakic (80–90%) area. The zonular fibres were lengthened in some area and were broken at places. Fundus examination was significant for tesselated appearance and supertraction crescent temporally. She was not cooperative for gonioscopy, intraocular pressure measurements, and forced duction test. Dynamic retinoscopy revealed absence of accommodation.

Systemic examination was significant for tall and thin posture with a reduced upper-to-lower segment ratio, increased arm-to-height ratio, arachnodactyly, positive wrist and thumb sign, hypermobility of joints (she could touch back of her thumb and the fingers to her forearm), and pes planus feet. She had a narrow face with highly arched palates and crowding of teeth. Cardiologic examination revealed no abnormalities. Genetic testings could not be done because of financial constraints. She was prescribed executive bifocal glasses and periodic checkup.

## 3. Discussion

Marfan syndrome (MFS) is an autosomal dominant disorder of connective tissue due to mutation of the fibrillin gene on chromosome 15q21.1 [1]. It is associated with characteristic skeletal, cardiovascular, and ocular manifestations [3, 4]. It affects both sexes equally and has no racial predilection. The incidence of MFS is estimated as 1 in 10,000 [3]. According to Ghent criteria [4, 5], an MFS patient must meet major criteria in 2 systems and have involvement of at least 1 other system (skeletal, cardiovascular, ocular) if family history is negative or unknown. Our patient did not have family history (sporadic case), but significant ocular and skeletal findings of MFS were present.

The DRS is a CCDD associated with anomalous innervation of the lateral rectus muscle by the oculomotor nerve [6]. Most cases of DRS are sporadic, but about 5–10% show autosomal dominant inheritance [2]. It is more common in females (60%), and the left eye appears to be affected more (60%). The incidence of DRS is <5% among the patients with strabismus [7]. The DRS is characterised by severe limitation of abduction and/or adduction or both. The genetic locus for type 1 DRS is mapped to 8q13 and type 2 is mapped to 15q21.1 [2, 8].

Our patient had a face turn, limited abduction and adduction in the left eye, and absence of convergence. However, other features of DRS such as globe retraction, palpebral aperture changes, and upshoot or downshoot were absent. Forced duction test, force generation test, and force degeneration test could not be performed. An electrophysiological diagnosis with electromyography and neuroimaging of abducens nerve with MRI of the brain was not possible due to financial constraints.

Rozen et al. [9] had reported a case of Marfanoid hypermobility syndrome associated with DRS. A Marfanoid hypermobility syndrome is different from MFS. Marfanoid hypermobility syndrome is an inherited connective tissue disorder with characteristics of MFS and Ehler-Danlos syndrome, in which the patient will show a very marked joint hypermobility and excessive stretchability of the skin. We did not find hyperextensibility of skin in our patient.

It has been reported that 30% of patients with DRS are associated with some systemic conditions which include Goldenhar syndrome, Klippel-Feil syndrome, Wildervanck syndrome, and congenital labyrinthine deafness [10]. To our knowledge, finding DRS on a typical MFS is uncommon and has not been reported before. Although both can be transmitted genetically as autosomal dominant trait, the genetic loci are on different chromosomes. We believe that the presentation of MFS with DRS in this patient was purely coincidental.

Literature search: Pubmed and Google search with key words as Marfan syndrome, Duane retraction syndrome for English literature.
